# Supplementary material for: An inverse agonist of orphan receptor GPR61 acts by a G protein-competitive allosteric mechanism
Source: Nat Commun. 2023 Sep 23;14:5938. doi: 10.1038/s41467-023-41646-3 (PMC10517971; doi:10.1038/s41467-023-41646-3)

Supplementary Information

Supplementary Methods

Compound 1 Selectivity Analyses:

GPCR selectivity panel:

Adrenergic alpha1a, dopamine 1, histamine 1, muscarinic 1, muscarinic 3 and serotonin 2b receptor activities were measured using FLIPR® Calcium Assays.

Cells used in the assay were stably transfected with the receptor of interest (adrenergic alpha1a, dopamine 1, histamine 1, muscarinic 1, muscarinic 3 and serotonin 2b). Activation of the receptor by an agonist in this assay system results in an increase in intracellular calcium levels which is measured using a calcium specific dye. Cells were plated at 7,500 cells per well (50 µL per well) in black walled clear bottomed 384-well plates 24 h prior to running the assay. Medium was removed from the plates and 80 µL of Hanks balanced salt solution (HBSS)/HEPES containing Calcium 5 dye (Molecular Devices, Sunnyvale CA, USA; Cat # R8186) and probenecid (1.25 mM) was added to each well and the plate was returned to the incubator for 1 h to allow dye loading. Compound solution (10 µL) was added to each well by the FLIPR Tetra® instrument (Molecular Devices, Sunnyvale CA, USA) to measure agonist activity of the compound by measuring the change in fluorescence from baseline over a 60 second period (Excitation 470-495 nm; Emission 515-575 nm). Subsequently 10 µL of agonist (EC_{80} value) was added to each well by the FLIPR Tetra® instrument to evaluate antagonist activity, with the change in fluorescence from baseline being measured over a 60 second period.

Adrenergic beta 2, cannabinoid 1 and mu opioid receptor activities were measured using Beta-Arrestin Assays.

The beta-arrestin assay relies on enzyme fragment complementation with the respective stably transfected GPCR (adrenergic beta 2, cannabinoid 1 and mu opioid) being tagged with an inactive portion of the enzyme β-galactosidase and a co-transfected β-arrestin that is tagged with the complementary portion of β-galactosidase. Recruitment of β-arrestin to the GPCR, results in a functional enzyme that generates a chemiluminescent signal when substrate is added. Cells were plated at 5,000 cells per well (40 µL per well) in black walled clear bottomed 384-well plates 24 h prior to running the assay. Medium was removed from the plates. For agonist studies 15 µL of HBSS/HEPES containing compound was added to the cells and the plate was incubated at room temperature for 90 min. For antagonist studies 15 µL of HBSS/HEPES containing compound was added to the cells and was incubated for 15 min prior to the addition of 15 µL of an EC_{80} concentration of agonist. The plate was subsequently incubated at room temperature for 90 min. Both assays were terminated by addition of 15 µL of a Beta-Glo® solution (Promega). Following an additional 30 min incubation the luminescence of each well was measured to determine the level of receptor activation.

Amine Transporter Assays

The amine transporter assay measures the ability of compounds to inhibit the activity of the norepinephrine (NET) dopamine (DAT) or serotonin (SERT) transporters by measuring the real time uptake of a dye labeled amine. HBSS/HEPES containing compound (5 µL) was added to the wells of black walled clear bottomed 384-well plate. Transporter dye (25 µL) (Molecular Devices, Sunnyvale CA, USA; Cat # R8174) was added to each well. Finally, 15,000 cells (20 µL) stably expressing the amine transporter of interest were added to each well and the plate is incubated at

37°C for 30 min (DAT) or 60 min (NET and SERT). The plate is transferred to the FLIPR Tetra® instrument and the fluorescence of each well was measured (Ex 470-495 nM; Em 515-575 nM). The level of fluorescence measured directly relates to the level of uptake of the dye labelled amine, with a reduction in levels being related to an inhibition of the respective transporter.

Phosphodiesterase Assays

The phosphodiesterase (PDE) assays measure the conversion of 3', 5'-[3H] cAMP to 5'-[3H] AMP (for PDE 3A1 and 4D3) or 3', 5'-[3H] cGMP to 5'-[3H] GMP (for 5A1) by the relevant PDE enzyme subtype. Yttrium silicate (YSi) scintillation proximity (SPA) beads bind selectively to 5'-[3H] AMP or 5'-[3H] GMP, with the magnitude of radioactive counts being directly related to PDE enzymatic activity. The assay was performed in white walled opaque bottom 384-well plates. Test compound (1 µL) in dimethyl sulfoxide was added to each well. Enzyme solution was then added to each well in buffer (in mM: Trizma, 50 (pH7.5); $MgCl_2$, 1.3 mM) containing Brij 35 (0.01% (v/v)). Subsequently, 20 µL of 3',5'-[3H] cGMP (125 nM) or 20 µL of 3',5'-[3H] cAMP (50 nM) was added to each well to start the reaction and the plate was incubated for 30 min at 25°C. The reaction was terminated by the addition of 20 µL of PDE YSi SPA beads (Perkin Elmer, Waltham, MA). Following an additional 8 h incubation period the plates were read on a MicroBeta radioactive plate counter (Perkin Elmer, Waltham, MA, USA) to determine radioactive counts per well.

Bromodomain-Containing Protein 4 (BRD4) Binding Assay

The BRD4 fluorescent polarization binding assay uses purified His-tagged BRD4 protein and its interaction with a Cy5 labelled small molecule probe that binds to the BRD4 site involved in the interaction with tetra-acetylated histone H4 peptide. In brief, the assay is performed in low volume

black 384 well flat-bottomed polystyrene plates. Compound/vehicle or standard (5 µL) were added to wells followed by His-tagged BRD4 (10 µL; 40 nM final concentration in assay). Following a 15 min incubation at room temperature a proprietary Cy5-labelled probe molecule (5 µL; 2 nM final concentration in assay) was added. Following, an additional 16 h incubation at room temperature fluorescence polarization measurements were made using an Envision plate reader (Perkin Elmer, Waltham, MA, USA) and mP values were used for analysis.

Acetylcholinesterase Assay

The assay described is based on Ellmans method, in which thiocholine produced by the action of acetylcholinesterase forms a yellow color with 5,5'-dithiobis(2-nitrobenzoic acid). The intensity of the product color, measured at 405 nm, is proportionate to the enzyme activity in the sample. To each well of a clear 96 polystyrene plate 90 µL enzyme solution (1mU/well) or phosphate buffered saline (PBS) and 10 µL compound/standard or vehicle was added. The plate was incubated at room temperature for 15 min. Subsequently 100 µL of substrate/detection reagent (800 µM acetylthiocholine/1mM 5,5'-dithiobis(2-nitrobenzoic acid)) was added and the plate was read at the 20 min time point.

hERG Binding Assay

Human embryonic kidney (HEK) cells stably transfected with a doxycycline inducible plasmid expressing the hERG channel (Accession Number: NM_000238) were cultured in suspension in Ex-cell 293 Serum Free Medium containing fetal bovine serum (5% v/v), L-Glutamine (6 mM), Blasticidin (5 µg/ml) and Zeocin (600 µg/ml) at 37 °C in a humidified environment (5% $CO2$/95% air). hERG expression was induced by the addition of doxycycline (1 µg/ml) 48 h prior to harvesting by centrifugation. Cell pellets were resuspended in ice cold homogenization buffer (1

mM EDTA, 1 mM EGTA, 1 mM NaHCO3, and cOmplete™ protease Inhibitor cocktail). Cells were homogenized using a dounce homogenizer (20 strokes), and centrifuged (1,000xg) for 10 min at 4ºC. The supernatant was transferred to a new tube and was centrifuged a second time (25,000xg) for 20 minutes at 4ºC. The supernatant was discarded, and the pellet was resuspended in buffer (50 mM HEPES, 10 mM MgCl2, bovine serum albumin (0.2% w/v) and cOmplete™ protease inhibitor cocktail). The samples were adjusted to 5 mg/ml and frozen. For the assay, membrane aliquots were thawed on ice and diluted to 200 µg/ml in assay buffer (25 mM HEPES, 15 mM KCl, 1 mM MgCl2, and 0.05% (v/v) Pluronic F127). A Cy3B tagged N-desmethyl dofetilide ligand was prepared in the same assay buffer solution (5 nM). Compound or vehicle (DMSO) was spotted into each well of a black 384-well low-volume plate. Membrane homogenate (15 µL) and Cy3B tagged ligand (10 µL) were then added to each well and the plate was incubated at room temperature for 16 h. Fluorescence polarization measurements were made using an Envision plate reader (Perkin Elmer, Waltham, MA, USA) and mP values were used for analysis. Binding Ki values were determined using the Cheng-Prusoff equation ($Ki = IC_{50}/(1+L/Kd)$), where L was the labelled ligand concentration in the assay (2 nM), and the Kd value (1.35 nM) the affinity constant for the labelled ligand.

hERG, Nav1.5 and Cav1.2 Ion Channel Profiling

Ionic currents were evaluated in the whole-cell configuration using the Qube384 automated planar patch clamp platform (Sophion Bioscience A/S, Ballerup, Denmark). QChip 384X plates, containing 10 patch clamp holes per well, were used to maximize success rate, which was routinely > 95%.

For hERG experiments, the external solution was composed of (in mM): 132 NaCl, 4 KCl, 1.8 $CaCl_2$, 1.2 $MgCl_2$, 10 HEPES, 11 Glucose, pH 7.4, 305 mOsM. The internal solution contained (in

mM): 15 NaCl, 60 KCl, 1 $MgCl_2$, 5 EGTA, 5 HEPES, 70 KF, pH 7.2, 300 mOsM. For Cav1.2 experiments, the external solution was composed of (in mM): 137.9 NaCl, 5.3 KCl, 0.49 $MgCl_2$, 10 $CaCl_2$, 10 HEPES, 0.34 Na_2HPO_4, 4.16 $NaHCO_3$, 0.41 $MgSO_4$, 5.5 glucose, pH 7.4, 310 mOsM. The internal solution contained (in mM): 27 CF, 112 CsCl, 2 $MgCl_2$, 10 EGTA, 10 HEPES, 2 Na_2ATP, pH 7.2, 305 mOsM. For Nav1.5 experiments (peak current), the external solution was composed of (in mM): 137.9 NaCl, 5.3 KCl, 0.49 $MgCl_2$, 1.8 $CaCl_2$, 10 HEPES, 0.34 Na_2HPO_4, 4.16 $NaHCO_3$, 0.41 $MgSO_4$, 5.5 glucose, pH 7.4, and osmolarity of 305 mOsM. The internal solution contained (in mM): 92 CsF, 55 CsCl, 2 $MgCl_2$, 5 EGTA, 5 HEPES, 1 MgATP, pH 7.2, 300 mOsM.

The hERG current was elicited from a holding potential of -80 mV by a voltage step to +40 mV for 500 ms, followed by a repolarizing ramp to -80 mV at -0.6 mV/ms. This pattern was repeated at a rate of 0.05 Hz. Peak hERG current was measured during the ramp. The Cav1.2 current was elicited by a voltage step to 0 mV for 150 ms from a holding potential of -40 mV. Voltage steps were repeated at 0.05 Hz, and Cav1.2 amplitude was measured as the peak current at 0 mV. For Nav1.5 current, from a holding potential of -80 mV, a 200 ms prepulse to -120 mV was used to homogenize channel inactivation, followed by a 40 ms step to a test potential of -15 mV. Membrane potential was further depolarized to +40 mV for 200 ms to completely inactivate the peak Nav1.5 current, followed by a ramp from +40 mV to -80 mV (-1.2 mV/ms). This voltage pattern was repeated at 0.1 Hz, with peak Nav1.5 defined as the maximum current during the step to -15 mV. All studies were conducted at 23° C.

Compounds were dissolved and initially diluted in dimethyl sulfoxide (DMSO), with a final dilution in external solution to generate final working concentrations. The final DMSO concentration in all experiments was 0.33% (v/v).

For all protocols three vehicle periods each lasting 5 minutes were applied to establish a stable baseline. For the standard protocol this was followed by the addition of increasing concentrations of test compound, with each exposure lasting 5 minutes. For the “extended” protocol following the three-vehicle additional each well subsequently received a single concentration of compound. This application was repeated three times for each well, via a flowthrough addition where the solution was replaced with the same compound concentration with each addition. Each exposure lasted 10 minutes.

Patch clamp data were analyzed using Assay Software 6.4.72 (Sophion Bioscience A/S, Ballerup, Denmark). Current amplitudes were determined by averaging the last 4 currents under each test condition. The percentage inhibition of each compound was determined by taking the ratio of current amplitude measured in the presence of various concentrations of the test compound (ICompound) versus the vehicle control current (IVehicle):

% Inhibition = [1-(ICompound/IVehicle)] * 100%.

A dose-response curve was generated and fit to the Hill equation by the Sophion Analyzer software to determine an IC_{50} value for each compound. The minimum and the slope of the fit were free fitted, with the top being fixed to 100% inhibition.

GABA Patch Clamp Assay

Compound effects on the human $GABA_A$ receptor (α1β2γ2), stably expressed in human embryonic kidney (HEK) cells, were examined in three modes of action: agonist, antagonist and positive allosteric modulation (PAM) modes. Chloride currents evoked by the activation of the $GABA_A$ receptor were recorded in the whole-cell patch clamp configuration with the automated Qube384® platform (Sophion Bioscience A/S, Baltorpvej, Denmark). The intracellular solution contained (in

mM): CsF 90, CsCl 50, $MgCl_2$ 2 EGTA 10, HEPES 10, pH adjusted to 7.2 with CsOH. The extracellular solution contained (in mM): NaCl 138, KCl 5.3, $CaCl_2$ 5, $MgCl_2$ 0.49, HEPES 10, glucose 5.5, Na_2HPO_4 0.34, $NaHCO_3$ 4.16, $MgSO_4$ 0.41, pH adjusted to 7.4 with NaOH. The osmolarity of the internal and external solutions were adjusted with sucrose to 300 mOsm and 305 mOsm, respectively.

Compounds were dissolved and initially diluted in dimethyl sulfoxide (DMSO), with a final dilution in external solution to generate final working concentrations. The final DMSO concentration in all experiments was 0.33% (v/v).

Cells and solutions were loaded into the Qube384 10X Qchip (10 recording wells per well, 384 wells per QChip). After whole-cell configuration was achieved by negative pressure pulse, cells were maintained at a holding potential of -80 mV throughout the experiment. To avoid desensitization and current rundown due to persistent activation of $GABA_A$, a "stacked pipette" approach was utilized to ensure only a brief exposure of the cells to GABA-containing solutions. In this approach, the perfusion pipettes drew from two liquid sources, first from extracellular solution (14 µL), and next from a GABA-containing solution (7 µL). When dispensed into the wells, this resulted in an exposure to GABA lasting 0.8 s, followed by immediate washout by extracellular solution. At the start of the experiment, a baseline $GABA_A$ current was established for each well in response to activation by 40 µM GABA. Each subsequent test condition was normalized against this baseline GABA current on a per well basis.

Agonist effects were measured by recording the current evoked by the test article in the absence of GABA. Agonism (%) was calculated relative to the current produced by 40 µM GABA for each well: [% Agonism = ($I_{\text{test article}}$ / $I_{40\mu\text{M GABA}}$) * 100%].

Antagonist effects of test article were examined in the presence of 40 µM GABA following a 4 min incubation in test article. Antagonism was calculated relative to the current produced by 40 µM GABA for each well [% Antagonism = ($I_{\text{test article, 40µM GABA}}$ / $I_{\text{40µM GABA}}$) * 100%]. Positive allosteric modulation (%) by test article was detected when current was enhanced relative to the 40 µM GABA normalization current.

EC_{50}/IC_{50} values were calculated by fitting concentration response curve data to a 4-parameter logistic regression equation [% effect=Bottom + (Top-Bottom)/(1+10^((LogIC_{50}-Concentration)*HillSlope))].

Data Analysis

Agonist/antagonist curves were plotted from individual experiments, and EC_{50}/IC_{50} values were determined using a four-parameter logistic fit. EC_{50} is defined as the concentration of the test article that produced a response that was equal to 50% of the maximal system response. IC_{50} is defined as the concentration of the test article that produced a 50% inhibition of a maximal response. An apparent K_B value for antagonist activity was calculated using the following equation:

Apparent K_B = IC_{50}/(1+([A]/Agonist EC_{50})

where the KB value is the dissociation constant of antagonist for the receptor, IC_{50} is the response produced by the test article in the presence of [A], the concentration of agonist used in the assay. Agonist EC_{50} is the EC_{50} value of the reference agonist used in the assay when tested alone.

Supplementary Materials

Supplementary Table 1: Compound 1 Selectivity. Stimulatory and/or inhibitory activity of compound 1 against a panel of drug safety target assays.

Supplementary Table 2: Compound 1 and HPE Compound Selectivity. Potency data for inhibition of GPR61, GPR62, and GPR101 by compound 1, as well as by a non-specific inhibitor used to define hundred-percent effect (HPE) for IC_{50} assays. SEM, 95% CI, and N are as indicated in the table.

Supplementary Table 3: Conformational Energetics of Compound 1. Select dihedral angle differences between the global minimum conformation of compound 1 and the bound cryo-EM conformation with estimated energy differences based on the OPLS4 force field.

Supplementary Table 4: Cryo-EM Data Collection, Processing, and Refinement Statistics.

Supplementary Note 1: Compound 1 Characterization Data. Measurements derived from ^{1}H, ^{13}C, and ^{19}F NMR spectra for Compound 1, as well as the calculated and measured mass from analysis by high-resolution mass spectrometry (HRMS).

Supplementary Figure 1: Expression and Constitutive Activity of GPR61 Mutants. a. Total cellular expression of wild-type GPR61 vs. point mutants in ECL2 and the TM6/7 disulfide. b. Cell surface expression of Hibit-tagged GPR61 wild-type and mutants as percentage of wild-type total expression. c. Cell surface expression of Hibit-tagged GPR61 wild-type and mutants as a percentage of wild-type cell surface expression. d. Basal cAMP activity of GPR61 mutants as a percentage of wild-type cAMP activity (untagged or Hibit-tagged). e. Cell surface expression-normalized basal cAMP activity of GPR61 mutants as a percentage of normalized wild-type cAMP activity (untagged or Hibit-tagged). For all panels, bar plots and error bars represent the mean ±

S.E.M. For panels a-c, N = 4 independent experiments. For panels d-e, N = 3 independent experiments. Asterisks indicate significance (*$p \leq 0.05$, **$p \leq 0.01$, ***$p \leq 0.001$), which was assessed using one-way ANOVA with one-sided Dunnett's post hoc test. Source data are provided as a Source Data file.

Supplementary Figure 2: Compound 1 Synthesis Scheme. The synthetic route used for the production of compound 1 is shown.

Supplementary Figure 3: AlphaFold-guided GPR61-BRIL Construct Design. a. General schematic for designing constructs. BRIL was fused, with or without linker sequences, to replace intracellular loop 3 of GPR61 for continouous helical extensions to GPR61 TM5 and TM6. b. General workflow for AlphaFold-based screening of constructs. AlphaFold predictions were used to bin construct designs based on the quality of helical fusions to TM5 and TM6 of GPR61. Predictions with relatively straight, helical fusions for both helices were selected for screening by cryo-EM. Cryo-EM screening enabled identification of a construct suitable for scale-up and full 3D reconstruction.

Supplementary Figure 4: Cell Surface Expression of GPR61 Wild-Type vs. BRIL Fusion. a. Schematic indicating how cells are binned based on their positions of the quadrants of Guava flow cytometry. Higher red fluorescence (y-axis) indicates cell death, while higher green (Alexa 488) fluorescence (x-axis) indicates higher receptor expression. Live fluorescent cells (lower right quadrant) are indicative of GPR61 cell surface expression. b. Guava results for GPR61-IA and GPR61 wild-type overexpressed in Sf9 insect cells. Column 1 represents cells in the absence of the anti-HA antibody, column 2 represents cells treated with the Alexa 488 antibody to indicate surface GPR61 expression level, and column 3 represents cells treated with the antibody and Triton X-100 to indicate total cellular GPR61 expression level. c. Percentage of live cells positive for

GPR61 cell surface expression with the two GPR61 constructs. d. Mean Alexa 488 fluorescence intensity for cell surface expression of the two GPR61 constructs. N = 1 independent experiment.

Supplementary Figure 5: Compound 1 Protein-Ligand Interactions. Residues proximal to compound 1 in the cryo-EM structure are indicated by labels. Green arrows indicate hydrogen bonds, with the arrow pointing from donor to acceptor.

Supplementary Figure 6: Basal Activity and Surface Expression of Compound 1 Binding Site Mutants. a. Relative basal activity of untagged vs. HiBit-tagged GPR61 WT and mutants. b. Relative surface expression of GPR61 WT and mutants. Bar plots and error bars represent the mean ± S.E.M. N = 3 independent experiments. Statistical significance is indicated with asterisks (*$p \leq 0.05$, **$p \leq 0.01$, ***$p \leq 0.001$) and was assessed using one-way ANOVA with one-sided Dunnett's post hoc test. Source data are provided as a Source Data file.

Supplementary Figure 7: Compound 1 Energy Calculations. a. Energy profile of the difluoropyridyl tail dihedral angle 1. Dihedral angles of the global minimum conformation (236.0°) and the cryo-EM conformation (255.3°) are indicated by the solid and dashed lines, respectively. b. 2D energy profile of the amine dihedral angles 2 and 3. The magenta circle indicates the global minimum conformation (74.9° and 0.3°, respectively) and the gray star indicates the cryo-EM conformation (122.1° and 330.2°, respectively). c. Energy profile of the sulfonamide dihedral angle 4. Dihedral angles of the global minimum conformation (125.9°) and the cryo-EM conformation (106.7°) are indicated by the solid and dashed lines, respectively.

Supplementary Figure 8: Multiple Sequence Alignment of Receptors Used for Selectivity Analyses and Other Biogenic Amine Receptors. Gene names of receptors used in selectivity analyses are underlined in green and GPR61 is underlined in magenta. Residues of GPR61 making

key polar interactions with Compound 1 are highlighted on the alignment by red boxes. Other residues lining the Compound 1 binding pocket are highlighted by blue boxes. Multiple sequence alignment was generated using ClustalW and visualization was created using BoxShade v.3.3.

Supplementary Figure 9: Cryo-EM Data Processing Workflow for Active-State GPR61-dnGαs/i chimera-Gβγ. a. Data processing workflow. b. Fourier shell correlation curves from gold-standard refinement and particle angular distribution. c. Cryo-EM map colored by local resolution. d. GPR61 TM helices fitted into the cryo-EM map.

Supplementary Figure 10: Cryo-EM Data Processing Workflow for $GPR61_{IA}$ + Compound 1. a. Data processing workflow. b. Fourier shell correlation curves from gold-standard refinement and particle angular distribution. c. Cryo-EM map colored by local resolution. d. GPR61 TM helices fitted into the cryo-EM map. e. Fitted model of compound 1 in its binding site with map density from two vantage points. Map is contoured at 5σ.

Supplementary Figure 11: Cryo-EM Data Processing Workflow for Apo-$GPR61_{IA}$. a. Data processing workflow. b. Fourier shell correlation curves from gold-standard refinement and particle angular distribution. c. Cryo-EM map colored by local resolution. d. GPR61 TM helices fitted into the cryo-EM map.

Supplementary Table 1

Compound 1 GPCR Selectivity		
**GPCR Target**	**Agonist EC50 (nM)**	**Antagonist IC50 (nM)**
**Alpha adrenergic receptor 1a**	>10,000	>10,000
**Beta-2 adrenergic receptor**	>10,000	>10,000
**Cannabinoid receptor 1**	>10,000	>10,000
**Dopamine receptor 1**	>10,000	>10,000
**Histamine receptor 1**	>10,000	>10,000
**Mu opioid receptor**	>10,000	>10,000
**Serotonin receptor 2b**	>10,000	>10,000
**Muscarinic receptor 1**	>10,000	>10,000
**Muscarinic receptor 3**	>10,000	>10,000

Transporter Targets	IC50 (nM)
**Serotonin Transporter**	>10,000
**Norepinephrine Transporter**	>10,000
**Dopamine Transporter**	>10,000

Phosphodiesterase Targets	IC50 (nM)
**Human PDE1B1**	>40000
**Human PDE2A1**	>200000
**Human PDE3A1**	18357
**Human PDE4D3**	>35018
**Human PDE5A1**	154918
**Bovine PDE6**	>200000
**Human PDE7B**	6273
**Human PDE8B**	9071
**Human PED9A1**	>200000
**Human PDE10A1**	>40000
**Human PDE11A4**	>40000

Epigenetic Targets	IC50 (nM)
**BRD4 (Binding)**	>25000

Enzyme Targets	IC50 (nM)
**Acetylcholinesterase**	>50000

Ion Channel Targets	Patch IC50 (nM)	Binding Ki (nM)
**hERG**	52904	>80000

Ion Channel Targets	%Activation@10µM	%Inhibition@10µM	EC50 (nM)	PAM EC50 (nM)
**GABAa**	0	4	>100000	>100000

Ion Channel Targets	IC50 (nM)
**Calcium Channel Cav1**	44787
**Sodium Channel Nav1**	>100000
**Sodium Channel Nav1.5 (Peak)**	>100000

	Period		
**Ion Channel Extended Protocol IC50 (nM)**	**1st**	**2nd**	**3rd**
**hERG**	36519	28050	18705
**Cav1.2**	21061	10729	5926
**Nav1.5 (Peak)**	>100000	>100000	>100000

Supplementary Table 2

		GPR61	GPR62	GPR101
**Compound 1**	**IC_{50} (µM)**	0.021	17.795	>29.741
	**95% confidence interval**	0.013-0.036	6.487-48.809	>29.085->30.412
	**Std Dev**	1.654	7.071	1.021
	**N**	6	4	6
**HPE Compound**	**IC_{50} (µM)**	1.896	1.569	1.863
	**95% confidence interval**	1.722-2.087	0.863-2.852	1.683-2.063
	**Std Dev**	1.901	1.767	1.946
	**N**	175	6	168

Supplementary Table 3

Dihedral Angle Name	Cryo-EM Dihedral Angle (°)	Global Minimum Dihedral Angle (°)	ΔG (kcal/mol)
Difluoropyridyl tail torsion **1** (NH-C-C-CF)	255.2	236.0	0.6
Amine torsion **2**	122.1	74.9	4.9
Amine torsion **3**	330.2	0.3	
Sulfonamide torsion **4**	106.7	125.9	2.2

Supplementary Table 4. Cryo-EM data collection, refinement and validation statistics

	GPR61-dnGαs/i chimera-Gβγ<br>(EMD-41144)<br>(PDB 8TB0)	GPR61-BRIL+ Compound 1<br>(EMD-41145)<br>(PDB 8TB7)	GPR61-BRIL apo
**Data collection and processing**			
Magnification	215,000x	215,000x	215,000x
Voltage (kV)	300	300	300
Electron exposure (e–/Å^2)	50	50	50
Defocus range (μm)	-2.4 to -0.6	-2.4 to -0.6	-2.4 to -0.6
Pixel size (Å)	0.59	0.59	0.59
Symmetry imposed	C1	C1	C1
Initial particle images (no.)	2,768,397	4,736,846	2,955,528
Final particle images (no.)	52,887	170,451	92,842
Map resolution (Å)<br>FSC threshold	3.47<br>(0.143)	2.90<br>(0.143)	3.97<br>(0.143)
Map resolution range (Å)		5.85-2.60	
**Refinement**			
Initial model used (PDB code)	AF_AFQ9BZJ8F1,<br>3SN6	AlphaFold,<br>6WW2	AlphaFold,<br>6WW2
Model resolution (Å)<br>FSC threshold	3.8<br>0.5	3.1<br>0.5	4.0<br>0.5
Model resolution range (Å)			
Map sharpening *B* factor (Å^2)	82.7	100.5	103.1
Model composition<br>Non-hydrogen atoms<br>Protein residues<br>Ligands	 8,467<br>1,090<br>0	 6,809<br>882<br>1	 6,304<br>399<br>0
B factors (Å^2)<br>Protein<br>Ligand	 65.52<br>N/A	 61.66<br>89.02	 234.12<br>N/A
R.m.s. deviations<br>Bond lengths (Å)<br>Bond angles (°)	 0.003<br>0.648	 0.003<br>0.613	 0.034<br>1.147
Validation<br>MolProbity score<br>Clashscore<br>Poor rotamers (%)	 1.85<br>8.75<br>0.11	 1.65<br>5.42<br>0	 2.28<br>23.32<br>0
Ramachandran plot<br>Favored (%)<br>Allowed (%)<br>Disallowed (%)	 94.37<br>5.63<br>0	 94.76<br>5.24<br>0	 93.70<br>6.05<br>0.25

Supplementary Note 1

Compound 1 Characterization Data

^{1}H NMR (400 MHz, DMSO-d_6) δ ppm 8.47 (s, 2H), 8.17 (br s, 1H), 7.89 (d, *J* = 8.4 Hz, 1H), 6.26 (s, 1H), 6.21 (d, *J* = 8.4 Hz, 1H), 4.63 (d, *J* = 6.1 Hz, 2H), 4.21 (t, *J* = 7.1 Hz, 2H), 4.11 (q, *J* = 6.9 Hz, 2H), 3.79 (s, 3H), 3.63 (t, *J* = 7.1 Hz, 2H), 2.18 (s, 3H), 1.18 (t, *J* = 7.1 Hz, 3H).

^{13}C NMR (101 MHz, DMSO-d_6) δ ppm 168.95, 167.69, 159.45, 159.24, 157.42 (dd, *J* = 259.78, 3.27 Hz), 157.13, 141.87, 134.14 (dd, *J* = 22.2, 6.1 Hz,) 123.38 (t, *J* = 15.2 Hz), 108.16, 100.18, 99.60, 70.29, 62.14, 58.05, 53.29, 45.73, 32.60, 23.27, 14.00.

^{19}F NMR (377 MHz, DMSO-d_6) δ –129.57.

HRMS calculated for $C_{22}H_{27}F_2N_6O_5S$ $[M+H]^+$ 525.1726, found 525.1715.

Supplementary Figure 1

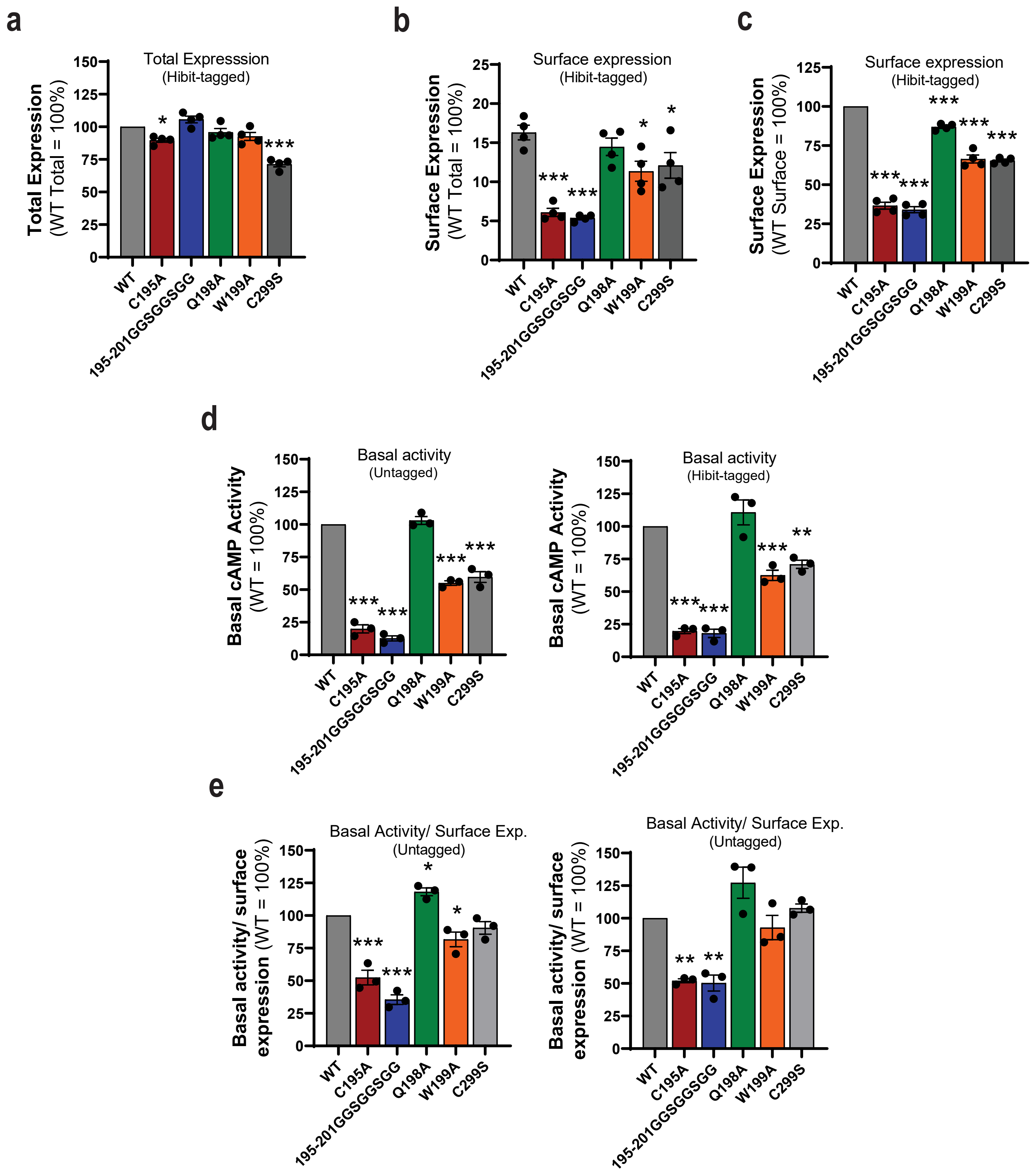

Supplementary Figure 2

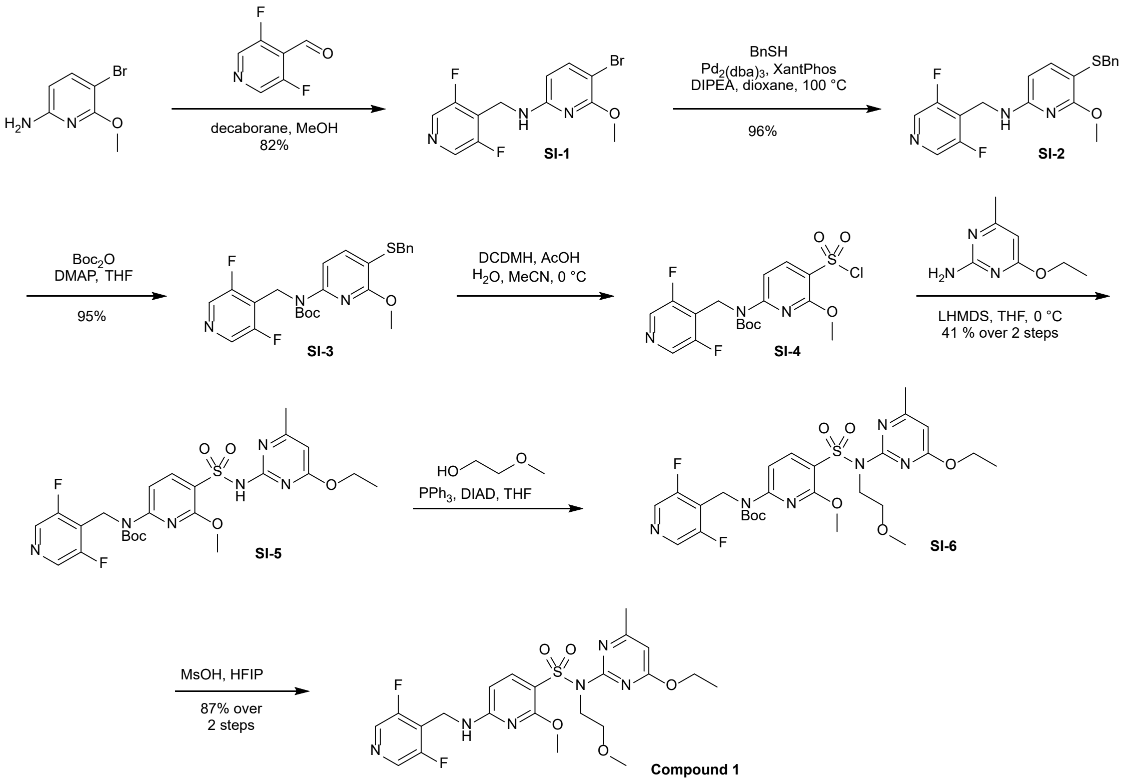

Supplementary Figure 3

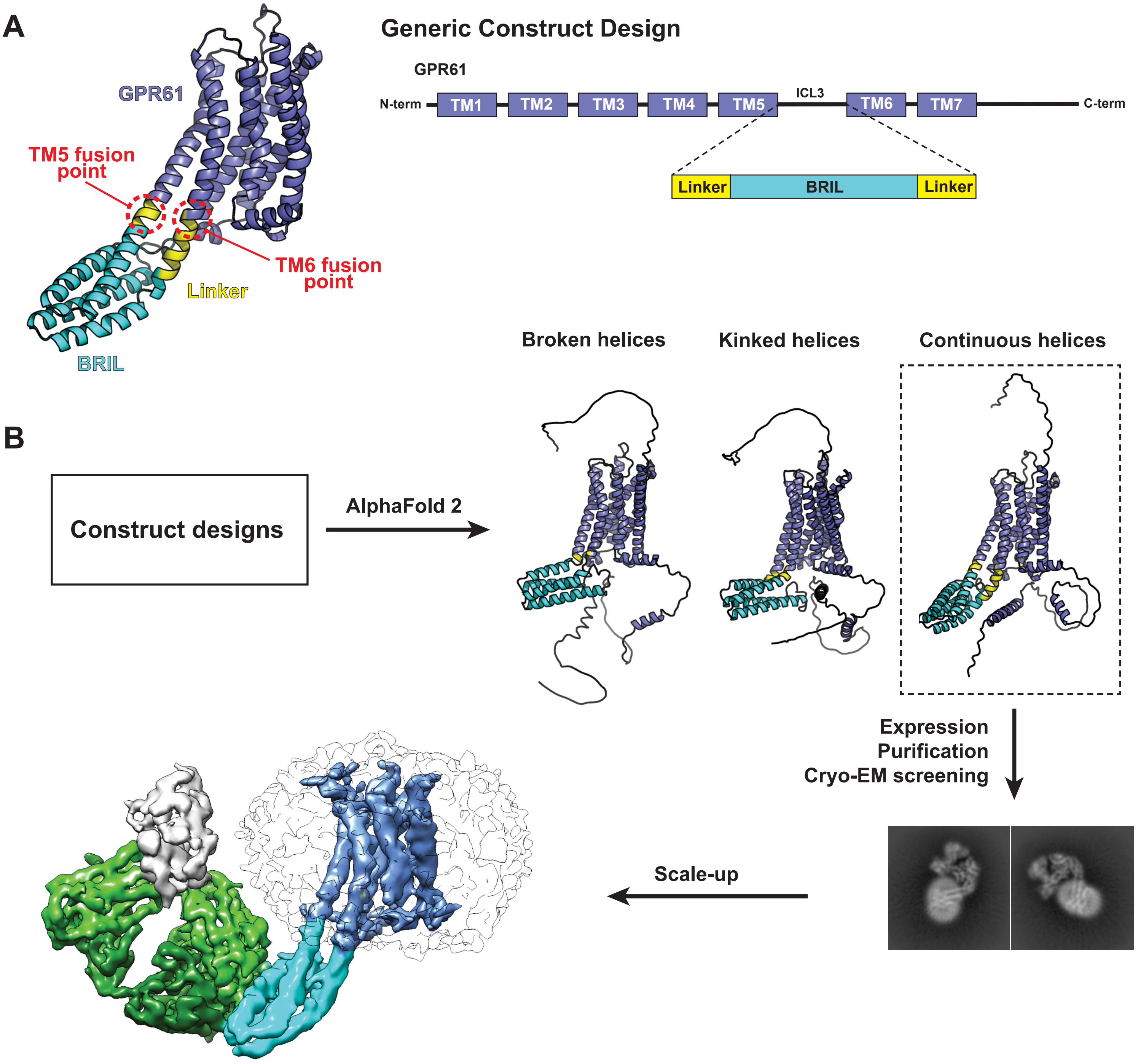

Supplementary Figure 4

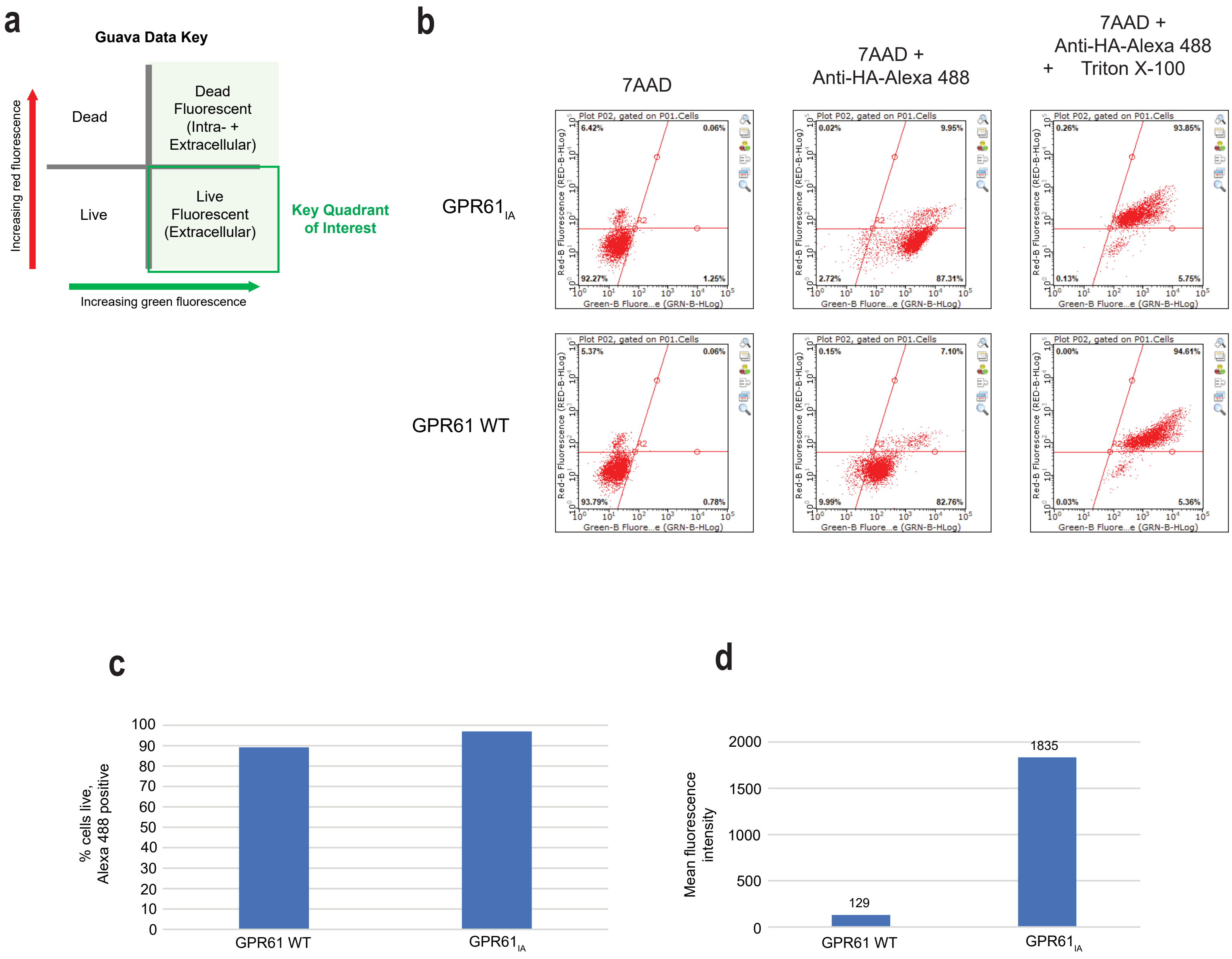

Supplementary Figure 5

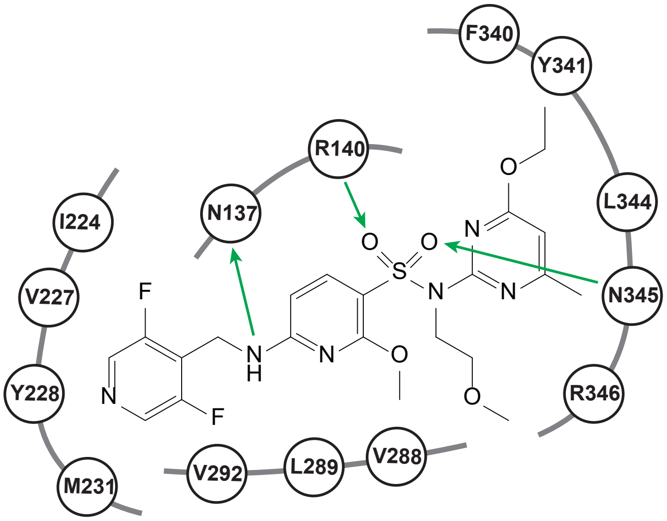

Supplementary Figure 6

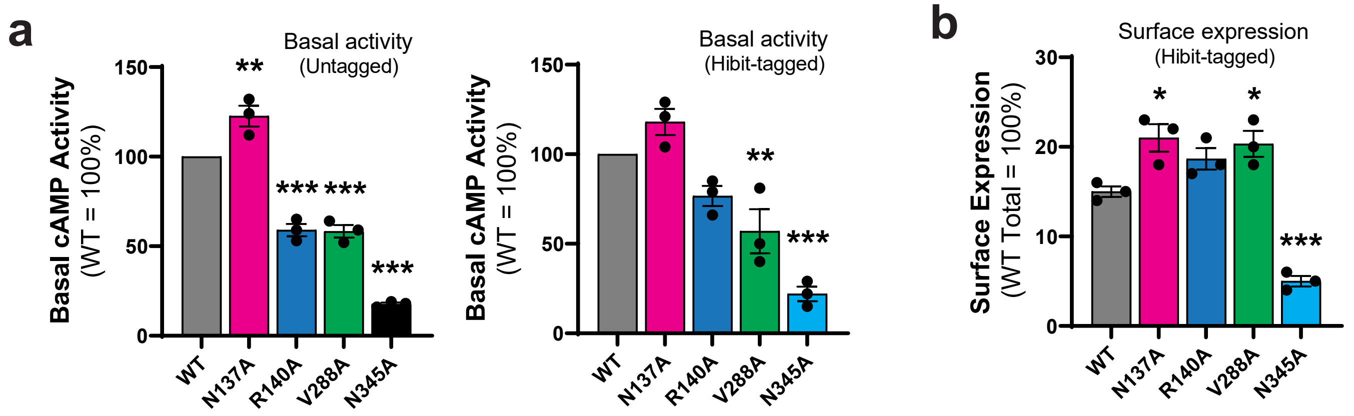

Supplementary Figure 7

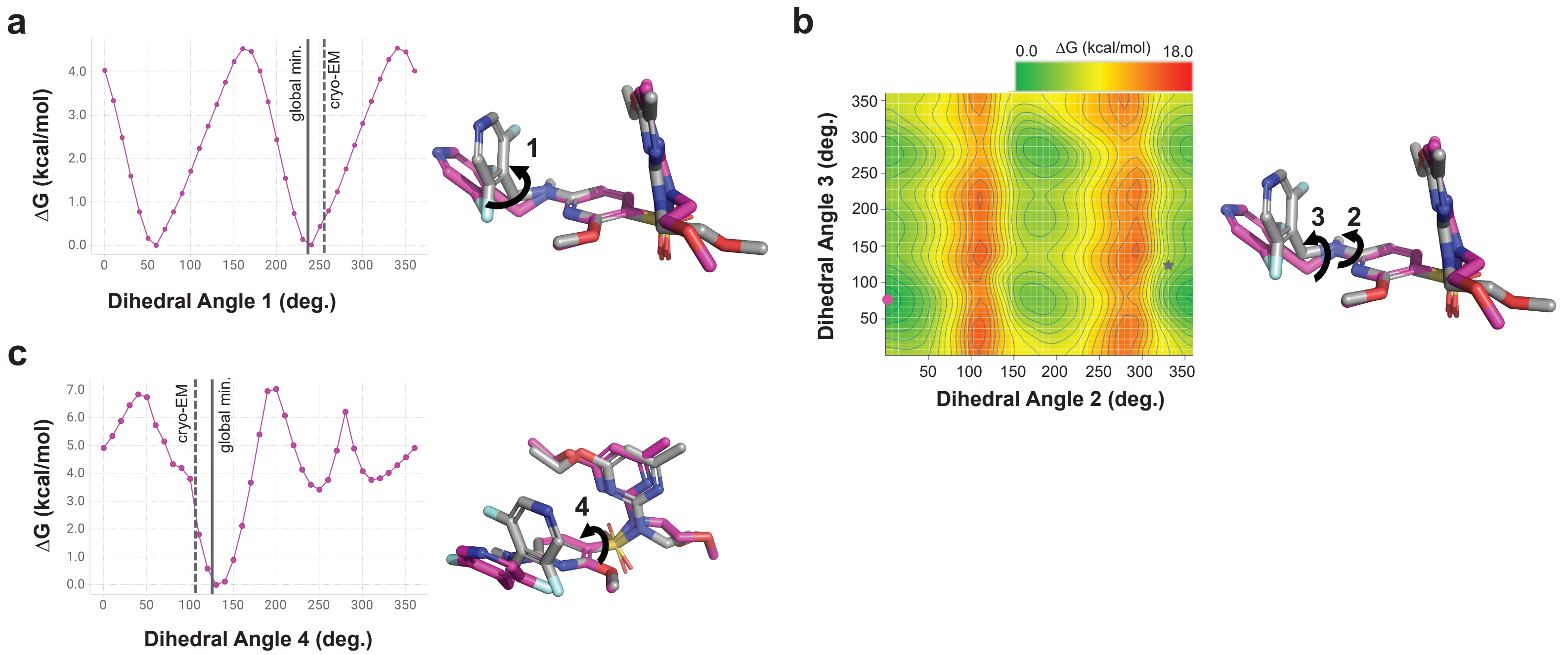

Supplementary Figure 8

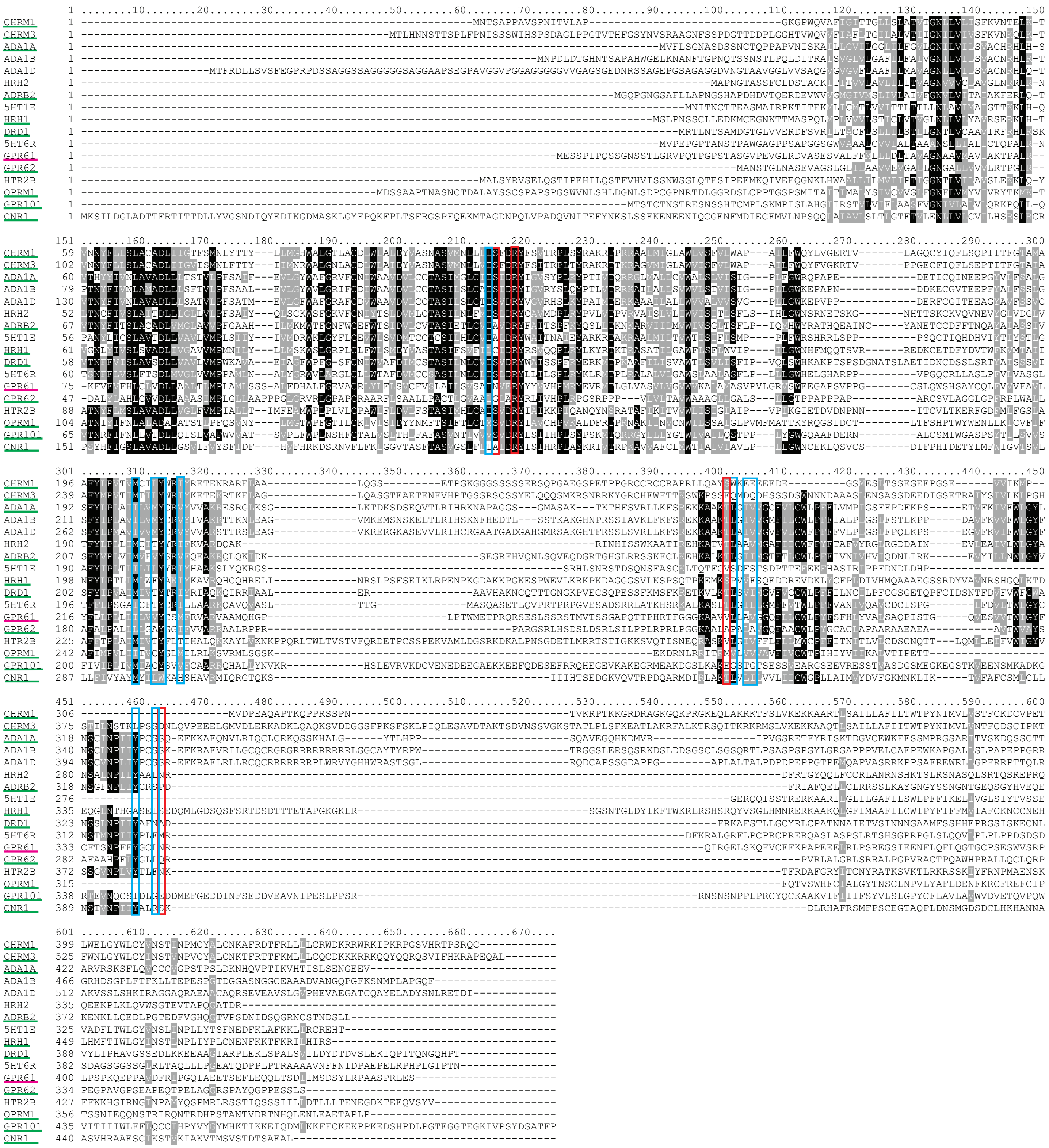

Supplementary Figure 9

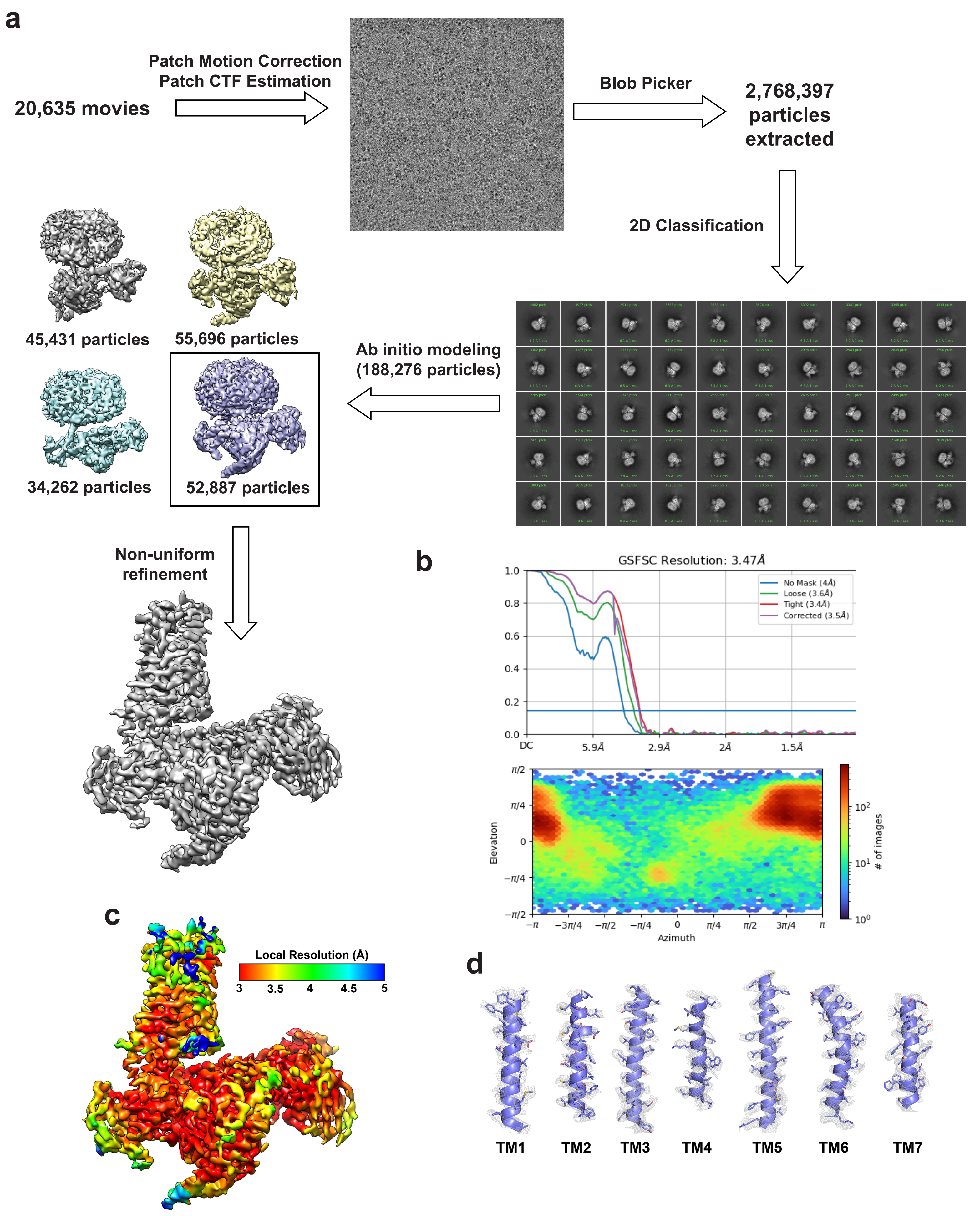

Supplementary Figure 10

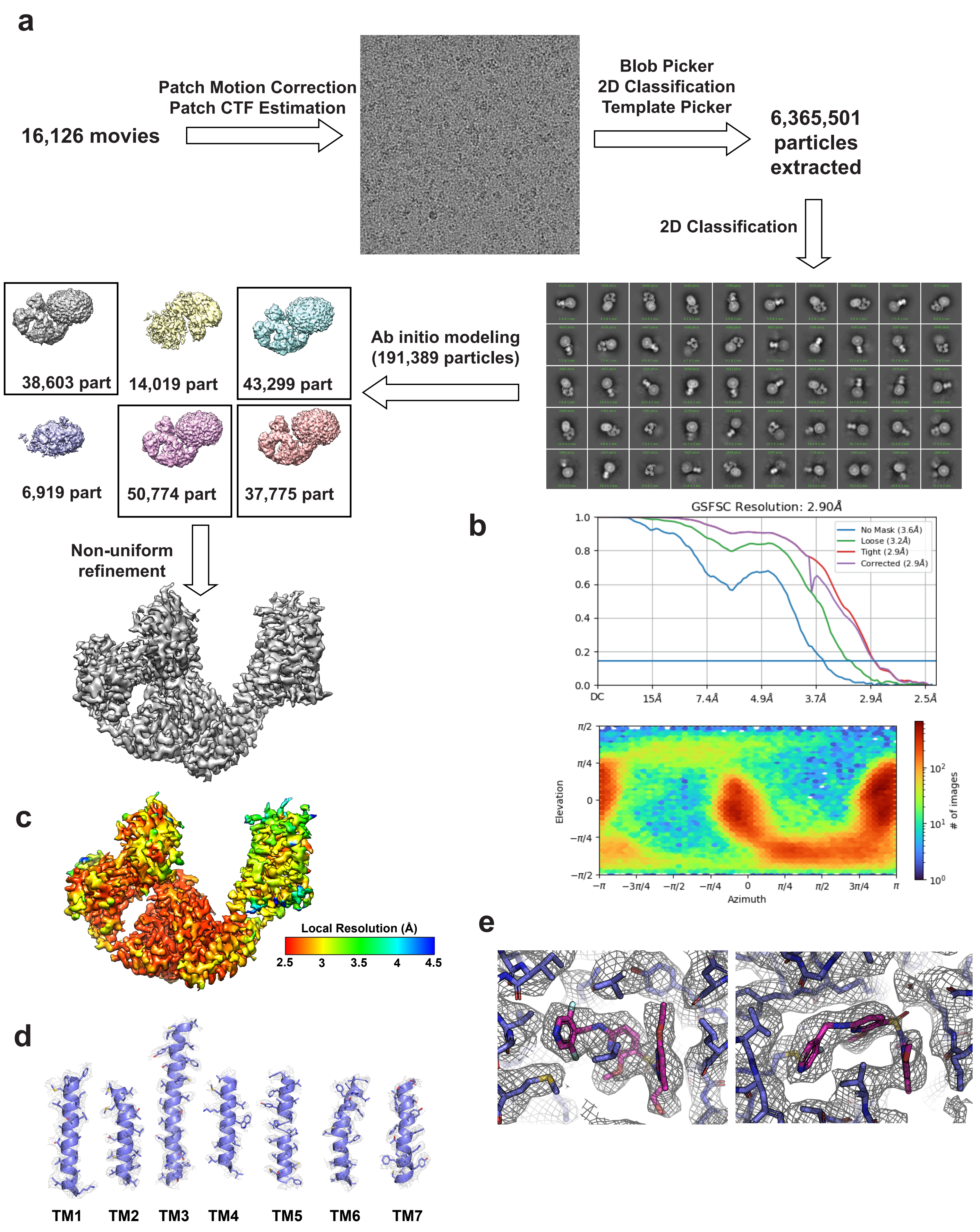

Supplementary Figure 11

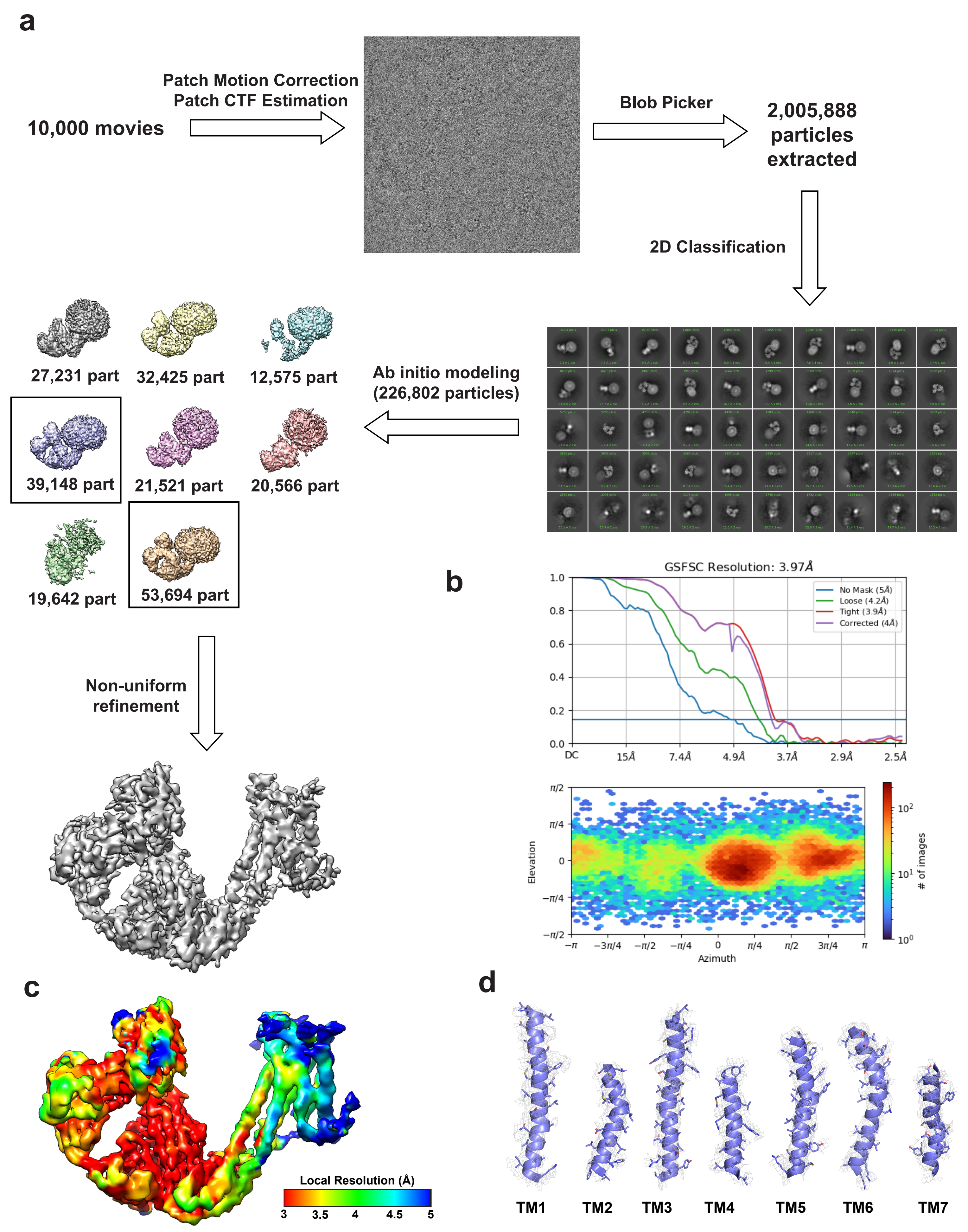

Supplement: Supplementary file 1 — Supplementary Information [file 41467_2023_41646_MOESM1_ESM.pdf]
