# Supplementary material for: An inverse agonist of orphan receptor GPR61 acts by a G protein-competitive allosteric mechanism
Source: Nat Commun. 2023 Sep 23;14:5938. doi: 10.1038/s41467-023-41646-3 (PMC10517971; doi:10.1038/s41467-023-41646-3)
Supplement: Supplementary file 3 — Reporting Summary [file 41467_2023_41646_MOESM3_ESM.pdf]

nature portfolio

Corresponding author(s): Seungil Han

Last updated by author(s): August 18, 2023

Reporting Summary

Nature Portfolio wishes to improve the reproducibility of the work that we publish. This form provides structure for consistency and transparency in reporting. For further information on Nature Portfolio policies, see our Editorial Policies and the Editorial Policy Checklist.

Statistics

For all statistical analyses, confirm that the following items are present in the figure legend, table legend, main text, or Methods section.

n/a	Confirmed	
☐	☒	The exact sample size (*n*) for each experimental group/condition, given as a discrete number and unit of measurement
☐	☒	A statement on whether measurements were taken from distinct samples or whether the same sample was measured repeatedly
☐	☒	The statistical test(s) used AND whether they are one- or two-sided<br>*Only common tests should be described solely by name; describe more complex techniques in the Methods section.*
☒	☐	A description of all covariates tested
☒	☐	A description of any assumptions or corrections, such as tests of normality and adjustment for multiple comparisons
☐	☒	A full description of the statistical parameters including central tendency (e.g. means) or other basic estimates (e.g. regression coefficient) AND variation (e.g. standard deviation) or associated estimates of uncertainty (e.g. confidence intervals)
☐	☒	For null hypothesis testing, the test statistic (e.g. *F*, *t*, *r*) with confidence intervals, effect sizes, degrees of freedom and *P* value noted<br>*Give P values as exact values whenever suitable.*
☒	☐	For Bayesian analysis, information on the choice of priors and Markov chain Monte Carlo settings
☒	☐	For hierarchical and complex designs, identification of the appropriate level for tests and full reporting of outcomes
☒	☐	Estimates of effect sizes (e.g. Cohen's *d*, Pearson's *r*), indicating how they were calculated

Our web collection on statistics for biologists contains articles on many of the points above.

Software and code

Policy information about availability of computer code

Data collection	EPU v3.2 was used for cryo-EM data collection
Data analysis	We used CryoSPARC v3.3.1, UCSF Chimera v1.16, ChimeraX v1.4, Coot v.0.9.8.1, Phenix v1.20, and PyMol v2.5.4 for cryo-EM data processing, modeling, and analysis. Schrödinger 2021-2 was used for computational chemistry calculations. GraphPad Prism v9.5.1 was used for assay data analysis.

For manuscripts utilizing custom algorithms or software that are central to the research but not yet described in published literature, software must be made available to editors and reviewers. We strongly encourage code deposition in a community repository (e.g. GitHub). See the Nature Portfolio guidelines for submitting code & software for further information.

Data

Policy information about availability of data

All manuscripts must include a data availability statement. This statement should provide the following information, where applicable:

- Accession codes, unique identifiers, or web links for publicly available datasets
- A description of any restrictions on data availability
- For clinical datasets or third party data, please ensure that the statement adheres to our policy

The cryo-EM maps generated in this study have been deposited in the Electron Microscopy Data Bank under accession codes EMD-41144 [https://www.ebi.ac.uk/emdb/EMD-41144] (GPR61-G protein complex structure) and EMD-41145 [https://www.ebi.ac.uk/emdb/EMD-41145] (GPR61 structure with compound 1). The

atomic coordinates corresponding to the cryo-EM maps generated in this study have been deposited in the Protein Data Bank under accession codes 8TB0 [https://doi.org/10.2210/pdb8TB0/pdb] (GPR61-G protein complex structure) and 8TB7 [https://doi.org/10.2210/pdb8TB7/pdb] (GPR61 structure with compound 1). Source data are provided with this paper.

Human research participants

Policy information about studies involving human research participants and Sex and Gender in Research.

Reporting on sex and gender	Not applicable
Population characteristics	Not applicable
Recruitment	Not applicable
Ethics oversight	Not applicable

Note that full information on the approval of the study protocol must also be provided in the manuscript.

Field-specific reporting

Please select the one below that is the best fit for your research. If you are not sure, read the appropriate sections before making your selection.

☒ Life sciences ☐ Behavioural & social sciences ☐ Ecological, evolutionary & environmental sciences

For a reference copy of the document with all sections, see nature.com/documents/nr-reporting-summary-flat.pdf

Life sciences study design

All studies must disclose on these points even when the disclosure is negative.

Sample size	CryoEM particle sample size was not pre-determined by any statistical metrics, but is consistent with those of other similar reports in the literature. Data processing yielded high-resolution reconstructions of the targets, suggesting that a sufficient number of particle images was collected. Sample sizes for other experiments were determined based on standards for experimental cell biology, attempting to have a minimum of N = 3 biological replicates with sufficient reproducibility.
Data exclusions	No data were systematically excluded. During cryo-EM data processing, particles with low contrast or poor correspondence/alignment to the consensus model from standard data processing were excluded from the final map calculations in CryoSPARC v3.3.1. Details are provided in the methods. For all other experiments, no data were excluded.
Replication	No replication of cryo-EM data was attempted, because it is unnecessary. Our cryo-EM structures were calculated according to standard procedures and do not require replicates. Unless otherwise indicated, assays reported here were performed at least three times independently and all attempts at replication were successful, with the exception of one instance of cAMP experiments for the ECL2 mutants. This one failed replicate was due to a technical issue: an incubator was found to be at 0% CO_2, leading to minimal cAMP basal activity window at WT GPR61 and inability to detect the activity of inverse agonists.
Randomization	The cryo-EM dataset was randomly split into two halves which were refined independently in cryoSPARC (i.e. "gold-standard" refinement) according to standard procedures. Resolution was assessed by the Fourier shell correlation between the half-maps at the 0.143 threshold criterion, indicating the resolution which is reproducible from this dataset. For all other experiments, because we performed the experiments with defined genetic and technique background, there was no need for randomization.
Blinding	No blinding was used nor necessary during cryo-EM data collection or analysis. Cryo-EM particle assignment to half-sets and the corresponding resolution estimation were performed automatically by standard processing software packages. For all other experiments, investigators were not blinded during data collection or analysis. Lab scientists directly performed experiments and analyzed the data generated. Determinations of relevant pharmacology parameters are considered objective measurements that are not subject to bias, so the integrity of the results is not compromised when the study and unblinded analysis are performed.

Reporting for specific materials, systems and methods

We require information from authors about some types of materials, experimental systems and methods used in many studies. Here, indicate whether each material, system or method listed is relevant to your study. If you are not sure if a list item applies to your research, read the appropriate section before selecting a response.

Materials & experimental systems

n/a	Involved in the study
☐	☒ Antibodies
☐	☒ Eukaryotic cell lines
☒	☐ Palaeontology and archaeology
☒	☐ Animals and other organisms
☒	☐ Clinical data
☒	☐ Dual use research of concern

Methods

n/a	Involved in the study
☒	☐ ChIP-seq
☒	☐ Flow cytometry
☒	☐ MRI-based neuroimaging

Antibodies

Antibodies used

Single-chain Fv16 (scFv16) is an antibody that binds to the Gi/o heterotrimer. Its sequence was acquired from published papers (https://doi.org/10.1038/s41586-018-0219-7 and https://doi.org/10.1038/s41586-018-0241-9. Hinge-binding nanobody (CaptureSelect™ LC-kappa (Human) Affinity Ligand) was purchased from Thermo-Fisher (#1033270500); FAb24 BAK5 is an affinity-matured synthetic antibody that binds to the BRIL sequence. Its sequence was acquired from the published paper in which it was reported, https://doi.org/10.1038/s41467-020-15363-0.

Validation

For scFv16 and anti-BRIL Fab24 BAK5, validation of antigen binding was described in the published papers referenced above. Additional validation was provided by observation of binding to purified protein and high-resolution cryo-EM reconstruction of antibody complexes with GPR61 and G protein complexes by cryo-EM. Validation of the hinge binding nanobody was reported by the manufacturer, referencing the following published paper, https://doi.org/10.1016/j.jmb.2017.12.010, and was confirmed by our observation of antigen binding to purified protein and high-resolution cryo-EM reconstruction of its complex with Fab24 BAK5.

Eukaryotic cell lines

Policy information about cell lines and Sex and Gender in Research

Cell line source(s)

Sf9 cells used for protein expression for cryo-EM were obtained from Thermo Fisher (#11-496-015). The Flp-In-CHO cell line used to generate CHO Trex cell lines was purchased from Thermo Fisher (#R75807). CHO-K1 cells were purchased from Lonza (Catalog number no longer available) and Eurofins (#CYL3038). HEK cells were purchased from Charles River (#CTN6199).

Authentication

Authentication of cell lines was performed by the manufacturers and no further validation was performed by the authors of this study.

Mycoplasma contamination

Cell lines were tested for mycoplasma contamination by the manufacturers. With the exception of Sf9 cells, all cell lines were tested for mycoplasma contamination upon receipt from the manufacturer, with negative results. No further mycoplasma testing was performed by the authors of this study.

Commonly misidentified lines
(See ICLAC register)

None of the cell lines used in this study have been classified as commonly misidentified cell lines.
